# Secondary deterioration in patients with normal pressure hydrocephalus after ventriculoperitoneal shunt placement: a proposed algorithm of treatment

**DOI:** 10.1186/s12987-020-00180-w

**Published:** 2020-03-04

**Authors:** Pawel Gutowski, Sergej Rot, Michael Fritsch, Ullrich Meier, Leonie Gölz, Johannes Lemcke

**Affiliations:** 1 Department of Neurosurgery, Unfallkrankenhaus Berlin, Warener Straße 7, 12683 Berlin, Germany; 2 Department of Neurosurgery, Dietrich Bonhoeffer Klinikum, Neubrandenburg, Germany; 3 Department of Radiology and Neuroradiology, Unfallkrankenhaus Berlin, Berlin, Germany

**Keywords:** NPH, Non-responder, Deterioration, Shunt failure, Neurodegenerative disease

## Abstract

**Background:**

After ventriculoperitoneal shunt surgery for idiopathic normal pressure hydrocephalus (iNPH) with adjustable gravitational valves, a certain proportion of patients develop secondary clinical worsening after initial improvement of clinical symptoms. The aim of this study was to analyze this group of patients with secondary deterioration and to evaluate the performed shunt management.

**Methods:**

For this investigation, we retrospectively reviewed our NPH registry for patients included between 1999 and 2013 with a decrease by a minimum of two points in the Kiefer score in the first year of follow up and an increase of two points in the Kiefer score between the second and the fifth year after shunt surgery (secondary deterioration). Then, we analyzed the patient’s shunt management (adapting the valve pressure setting, shuntography, valve replacement, catheter replacement, implant an adjustable gravitational unit). Additionally, we searched for risk factors for secondary deterioration.

**Results:**

Out of 259 iNPH patients, 53 (20%) patients showed secondary deterioration on an average of 2.7 (2–4 years) years after shunt surgery. Fourteen (26%) patients with secondary deterioration improved after shunt or valve management and 58% remained without clinical benefit after management. We had a drop-out rate of 15% due to incomplete datasets. Our shunt management reduced the rate of secondary deterioration from 20 to 15%. On the basis of our findings, we developed an algorithm for shunt management. Risk factors for secondary deterioration are the age of the patient at the time of shunting, newly diagnosed neurodegenerative diseases, and overdrainage requiring adjusting the valve to higher-pressure levels.

**Conclusion:**

Twenty percent of patients with iNPH were at risk for secondary clinical worsening about 3 years after shunt surgery. About one-fourth of these patients benefited for additional years from pressure level management and/or shunt valve revision. Our findings underline the need for long-term follow-ups and intensive shunt management to achieve a favorable long-term outcome for patients with iNPH and VPS.

## Background

In 1965, Hakim and Adams [1] first described normal pressure hydrocephalus (NPH) which is characterized by a triad of symptoms with gait instability, urinary incontinence, and dementia. The incidence of iNPH in Germany is 1.08 per 100,000 [2]. Other studies reported an incidence of 1.19 to 5.5 per 100,000 [3, 4]. The prevalence of iNPH increases with age: 0.2% at 70–79 years and 5.9% at 80 years and older [5]. Tanaka et al. [6] considered a prevalence of iNPH in the elderly to be 1.4%.

The designation NPH is subdivided into idiopathic NPH (iNPH) and secondary NPH (sNPH) [7], where sNPH arises as a possible consequence of meningitis, encephalitis, traumatic brain injury or subarachnoid hemorrhage [8]. A meticulous preoperative diagnosis is crucial for a favorable response to VP shunting in patients with iNPH [8]. Common comorbidities among iNPH patients, arterial hypertension, diabetes mellitus, and vascular dementia, may be major prognostic factors [9]. Neurodegenerative diseases (dementia with Lewy bodies, Parkinson`s disease, Alzheimer’s disease, progressive supranuclear palsy, frontotemporal dementia) could also present with extrapyramidal symptoms mimicking some symptoms of NPH [10, 11]. Therefore, a comprehensive differential testing is required. Imaging shows pathologically enlarged ventricular size with an Evans index [12] (the ratio of the widest diameter of the frontal horns to the widest diameter of the brain on the same axial slice) of more than 0.3 [13, 14]. A corpus callosum angle of 40° or more and a lack of subarachnoid space over the high convexity (DESH-disproportionately enlarged subarachnoid space hydrocephalus) are among the key neuroradiological features [8], together with a CSF opening pressure ranging from 5 to 18 mmHg (or 70–245 mmH_2_O) [8]. Specific tests for iNPH include the lumbar infusion test, CSF drainage (tap test) by lumbar puncture or lumbar drain [15–21]. The results of the elaborate diagnostics correlate with the primary shunt response [21, 22].

The treatment of choice is ventriculoperitoneal (VP) shunt placement with adjustable valves [7] and about 74% of patients with iNPH benefit from VP shunting [22, 23]. Studies with long term follow up of iNPH patients after shunting suggest, that a number of patients show delayed deterioration of their symptoms despite an improvement in the first months [23, 24].

The aim of this study was to investigate iNPH patients from our register with secondary deterioration of their initially improved symptoms. The purpose was to find the number of patients with primary deterioration in the first year after VPS placement and also the number with primary improvement but followed by secondary worsening after VP shunting. Further, our goal was to determine clinical predictors for secondary worsening and to distinguish whether the deterioration is based on the progression of the hydrocephalus and/or co-morbidities, or associated with VP shunt malfunction. Based on our findings, we have also developed an algorithm for the standardization of treatment steps in order to further minimize the proportion of secondary non-responders.

## Materials and methods

In our Department of Neurosurgery all patients with the suspected diagnosis iNPH were diagnosed using a published diagnostic pathway including an intrathecal infusion test and a CSF tap test [23]. A positive result in the invasive diagnostic method is defined as: (1) resistance to outflow (R_out_) of 13 mmHg/min × mL or more in the lumbar CSF infusion test; (2) improvement of the performance of walking and turning by a minimum of 20% after spinal tap test. If only one test turns out positive, then a lumbar drain is placed for 3 days to measure the performance of walking and turning over this period of time. In the case of a positive result in two diagnostic methods, the patient is classified for VP shunting. After VP shunting all patients are regularly scheduled for follow up examinations at 3, 6 and 12 months and thereafter yearly. During the follow up examinations we scored the patients’ symptoms using the Kiefer score [25] (KS) for iNPH. The KS contains gait disorder (0–6 points), cognitive decline (0–6 points), urinary incontinence (0–6 points), headache (0–4 points), and vertigo (0–2 points). The symptoms were assigned according to their severity. Furthermore, we calculated the Evans indices from the recent cerebral computer tomographic (CT) scans at each follow-up. The comorbidity index (CMI) [26] describes the sum of the patients’ comorbidities. Hypertension, aortofemoral bypass, stent, ICA stenosis, posterior circulation insufficiency, arrhythmia, valvular disease, heart failure, aortocoronary bypass and infarction are calculated with one point, respectively. Diabetes mellitus, peripheral vascular occlusion, vascular encephalopathy, transient ischemic attack, prolonged reversible neurologic deficit and Parkinson’s disease are calculated by two points, respectively. The cerebral infarct is rated with three points. The collected data were entered in our iNPH registry file.

We retrospectively reviewed our iNPH registry. All patients in our registry underwent shunt surgery. Our iNPH registry was initiated in 1999 and is still ongoing. To determine the rate of iNPH patients with primary deterioration, we chose for review a period from 1999 until 2017. To investigate patients with secondary deterioration, we considered patients who were included in our registry until the end of 2013. This period allowed us to have enough follow-up time for analyzing the clinical course. Patients who showed equal or worse Kiefer scores in the first year of follow-up, compared to the preoperative score, were defined as “primary deteriorated”. For this study, we did not investigate this subgroup in detail.

“Secondary deterioration” was defined as a decrease by the minimum of two points in the Kiefer score in the first year of follow-up and an increase of two points in the Kiefer score between the second and the fifth year after shunt surgery. Patients with secondary deterioration resulting from a treatable mechanical shunt issue by decreasing the valve pressure setting, shuntography, and/or surgical shunt revision were defined as “shunt insufficiency”. Patients with secondary deterioration and unsuccessful shunt management (adapting the valve pressure setting, shuntography, valve replacement, catheter replacement, implantation of an adjustable gravitational unit) were assigned as “secondary non-responder”.

All patients included in this study had received adjustable pressure valves with fixed gravitational valves (proGAV, Aesculap-Miethke, Potsdam, Germany; Medos-Hakim, Codman and Shurtleff, Johnson and Johnson, Ryanham, Massachusetts, USA). Patients with implanted DualSwitch valves (Aesculap-Miethke, Potsdam, Germany) were not considered in this investigation. The initial pressure level of the adjustable unit was set to 70–100 mmH_2_O. At the 3 months follow-up, the valves were readjusted to 50–70 mmH_2_O. The decision to adjust the pressure was done because of several recommendations in literature suggesting better outcomes [27–29]. The decision for the pressure level of the fixed gravitational units was taken from the recommendation of the manufacturer (200–300 mmH_2_O).

In summary, the inclusion and exclusion criteria to the retrospective investigation of patients out of our iNPH registry were:

### Inclusion criteria

- Patients with iNPH (typical symptoms, radiomorphological findings, infusion- test, spinal-tap-test).
- Implantation of adjustable units or non- adjustable gravitational units (pressure level 50–100 mmH_2_O or 200–300 mmH_2_O, respectively).
- Decrease of the Kiefer score by a minimum of 2 points in the first year after shunt surgery.
- Increase of the Kiefer score by a minimum of 2 points between the second and the fifth year after shunt surgery.

### Exclusion criteria

- sNPH.
- Non-communicating hydrocephalus.
- Primary deterioration (pre-VP Shunting Kiefer score equal or better to Kiefer score in the first year of FU).
- Implanted shunt valves alone without shunt assistant.

In the second step, medical records of the included patients were screened. The medical management (adapting the valve pressure setting, shuntography, valve replacement, catheter replacement, implantation of an adjustable gravitational unit), performed to improve the symptoms of the patient, was evaluated. The medical records of the patients with secondary deterioration were screened for new diagnosed neurodegenerative diseases.

Statistical evaluation was performed using Prism for Mac OS (GraphPad) and Microsoft Excel for Windows (Microsoft Corp.). Differences between groups were tested using Chi-squared-test, Fisher’s exact test, Mann–Whitney test, Kruskal–Willis test and the t-test. Multivariate analysis of variance was performed. The level of significance was defined as p ≤ 0.05.

## Results

**Fig. 1:**
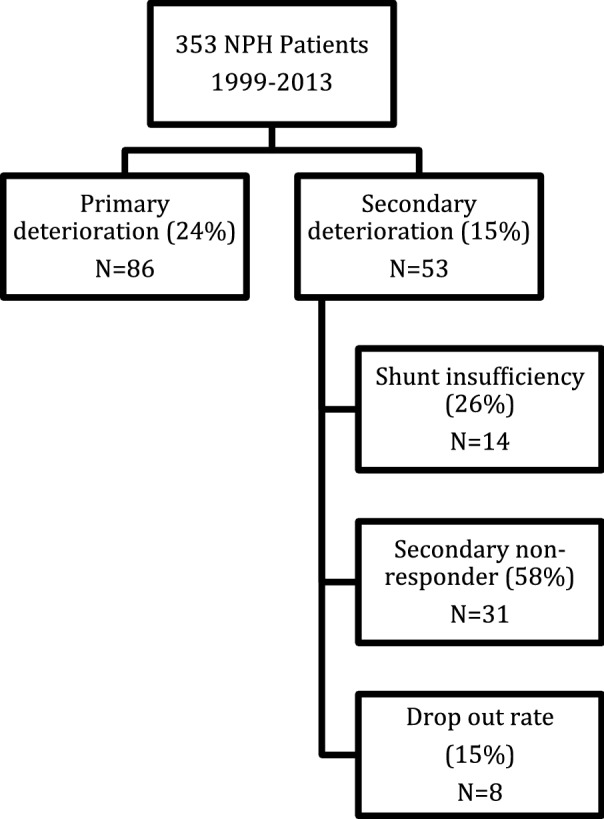
Flow diagram showing our study population of normal pressure hydrocephalus patients (NPH)

Between 1999 and 2017, 353 patients suffering from iNPH were surgically treated with VP Shunt in the Department of Neurosurgery of the Unfallkrankenhaus Berlin. Out of these 353 patients, 86 (24% of 353) patients showed “primary deterioration”. Eight patients (2.2% of 353) were lost to follow-up in the first 6 months. In the period of 1999 until 2013, we included 259 patients in our iNPH registry. Fifty-three patients (20% of 259) met our inclusion criteria (see Fig. 1). We observed secondary worsening on average 2.7 (2–4 years) years after shunt surgery. Out of this group, fourteen patients (26% of 53) showed a manageable shunt insufficiency and improved according to the Kiefer score and thirty-one patients (59% of 53) remained as secondary non-responders despite at least one action taken: decreasing the valve pressure setting, shuntography, valve replacement, catheter replacement, or implantation of an adjustable gravitational unit (Mann–Whitney U test, p = 0.004). Five (16%) of the thirty-one patients developed radio-morphological and/or clinical symptoms of overdrainage. Thus, the valve pressure had to be set to a higher opening pressure level (100–120 mmH_2_O) with the consequence of increasing the Kiefer score.

Out of the group with “secondary deterioration” (N = 53), we had a dropout rate of 15% (8 of 53) resulting from incomplete datasets for the follow up examinations. Although these eight patients showed secondary deterioration, the dataset of the follow-ups was not complete with regard to the medical management. We show the baseline data in Table 1. There were no significant differences between the respective groups. Interestingly, the comorbidity index (CMI) [26] at the time of surgery was similar in all groups.

**Table 1:** Baseline demographic characteristics of the patients

	Total (1999–2017) (N = 353)	Primary deterioration (N = 86)	Total (1999–2013) (N = 259)	Secondary deterioration (N = 53)	Shunt insufficiency (N = 14)	Secondary non-responder (N = 31)	Drop-outs (N = 8)	Test and p-value
Age (years) (at the time of surgery)								
Mean (median) ± SD	71.4 (72) ± 10	74 (72) ± 10	71 (72) ± 10	70 (72) ± 11	68.5 (68) ± 8	73.3 (72) ± 4.2	74.9 (77) ± 8.6	Kruskal–Wallis test p = ns
Sex								
Female n (%)	146 (42%)	40 (47%)	104 (40%)	22 (41%)	4 (28%)	15 (48%)	3 (38%)	Chi-square test p = ns
Male n (%)	207 (58%)	46 (53%)	155 (60%)	31 (59%)	10 (72%)	16 (52%)	5 (62%)	
Pre-VPS CMI								
Mean (median) ± SD	2.6 (3) ± 1.9	2.2 (2) ± 1.9	2.7 (3) ± 2	2.5 (2) ± 2	2.2 (3) ± 1.8	2.7 (2) ± 2.3	2.1 (1.5) ± 2.1	Kruskal–Wallis test p = ns
Kiefer score								
Mean (median) ± SD	7.1 (7) ± 2.9	6.1 (6) ± 2.7	7.6 (7) ± 3	6.8 (7) ± 2.7	7 (7) ± 3.6	6.8 (7) ± 2.3	6.9 (6.5) ± 2.2	Kruskal–Wallis test p = ns

*CMI* Comorbidity Index, *VPS* ventriculoperitoneal shunt

In Table 2, the respective accomplished medical management is depicted for patients with secondary deterioration. No statistically significant difference was found between the two groups “shunt insufficiency” and “secondary non-responder”.

**Table 2:** Shunt management of the patients with secondary deterioration with the exclusion of the patients with overdrainage

	Shunt insufficiency (N = 14)	Secondary non-responder (N = 26)	Test and p-value
Valve pressure (0–30 mmH_2_O) (N)	14	26	Fisher’s exact test p = ns
Shuntography (N)	8	14	Fisher’s exact test p = ns
Implantation of an adjustable ASD (N)	6	7	Fisher’s exact test p = ns
Catheter replacement (N)	0	0	

*ASD* anti-siphon-device

The baseline data of the patients with “shunt insufficiency” and the “secondary non-responders” (see Table 3) showed that the “secondary non-responders” were older at time of surgery than the patients with “shunt-insufficiency” (73 vs. 68.5 years; t-test, p-value = 0.01). The preoperative CMI is equal in both investigated groups (Mann–Whitney test, p = ns) and similarly in the preoperative Kiefer score (Mann–Whitney test, p = 0.10). Patients with a newly diagnosed neurodegenerative disease after VP shunting are overrepresented, but not statistically significant, in the group of “secondary non-responders” (Fisher’s exact test, p-value = ns). The presence of clinical and/or radio-morphological signs of overdrainage was noted exclusively in the subgroup of “secondary non-responder” (Fisher’s exact test, p-value = ns).

**Table 3:** Comparison of the two patient groups

	Shunt insufficiency (N = 14)	Secondary non-responder (N = 31)	Test and p-value
Age			
Mean (median) ± SD (at the time of surgery)	68.5 (68) ± 8	73.3 (72) ± 4.2	t-test p = 0.01
Pre-VPS CMI			
Mean (median) ± SD	2.2 (3) ± 1.8	27 (2) ± 2.3	Mann–Whitney test p = ns
Kiefer score			
Mean (median) ± SD	7 (7) ± 3.6	6.8 (7) ± 2.3	Mann–Whitney test p = ns
New diseases during the F/U (stroke, Alzheimer disease, Parkinson disease)	1 (7%)	6 (19%)	Fisher’s exact test p = ns
Over-drainage requiring valve pressure setting ≥ 100 mmH_2_O	0 (0%)	5 (16%)	Fisher’s exact test p = ns

*CMI* Comorbidity Index, *VPS* ventriculoperitoneal shunt, *F/U* follow-up

Following up our findings, we carried out a multivariate analysis of the risk factors. The multivariate analysis showed, that age is the strongest risk factor to become a “secondary non-responder” (p-value = 0.03). The preoperative CMI and Kiefer score have no significant impact as a risk factor (p-value = ns; p-value = ns). Overdrainage and newly diagnosed neurodegenerative diseases did not reach the significance level in the multivariate analysis (p-value = ns; p-value = ns).

We compared the outcome of shunt management between the shunt insufficiency and the secondary non-responder groups (Table 4). There was no statistically significant difference between the accomplished shunt management in these two groups. Although the results are not statistically significant, a more comprehensive shunt management (43% in the “shunt insufficiency” group vs. 26% in the “secondary non-responder” group) seems to lead to a reversible secondary deterioration by completing all three shunt management steps. Thus, our shunt-management decreased the rate of secondary deterioration in the total investigated study population from 20 to 15% (Fisher’s exact test, p = ns).

**Table 4:** Comparison of the shunt management as measured by improvement in the Kiefer score, of the shunt-insufficiency group and the group of secondary non-responders, after exclusion of the patients with overdrainage

	Shunt insufficiency (N = 14)	Secondary non-responder (N = 26)	Test and p-value
Valve pressure setting (N)	5 (36%)	12 (46%)	Fisher’s exact test p = ns
Valve pressure setting + shuntography (N)	3 (21%)	7 (27%)	Fisher’s exact test p = ns
Valve pressure setting + shuntography + implantation of an adjustable ASD (N)	6 (43%)	7 (27%)	Fisher’s exact test p = ns

*ASD* anti-siphon-device

Based on our results that comprehensive shunt management seems to show the opportunity for optimize the long-term outcome of iNPH patients. Over 64% (see Table 4) of the patients in “shunt insufficiency” group needed invasive shunt management with at least shuntography and finally the replacement of the fixed antisiphon device (ASD) to an adjustable device to improve the symptoms. In the group of “secondary non-responder”, 54% got invasive shunt management.

**Fig. 2:**
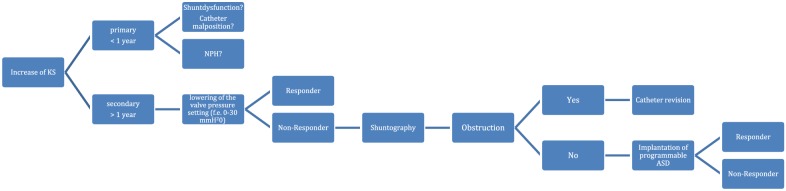
Algorithm for shunt management of patients with normal pressure hydrocephalus and ventriculoperitoneal shunt. Based on our findings, in 2018, we developed and established in our department this algorithm for management of normal pressure hydrocephalus patients to decrease the rate of “secondary non- responder”. *KS* Kiefer score, *NPH* normal pressure hydrocephalus

Therefore, we developed an algorithm of a complex treatment strategy of iNPH patients with deterioration after VPS placement with the purpose to decrease the rate of “secondary non-responder”. Figure 2 shows in detail our steps of management of these patients.

## Discussion

Our results reveal that 20% of the iNPH patients treated with VP shunting (VPS) deteriorated in their symptoms after an average of 2.7 years, despite a clinical benefit in the first year after the VPS. The observation, that a delayed deterioration occurred at an average of more than 2 years after VPS, is consistent with published studies [11, 30]. Twenty-six percent of the patients with secondary deterioration improved after shunt/valve management, 59% remained without clinical benefit after taking action of the VPS. The results of the “shunt insufficiency” group shows, that over 64% of the patients require a more complex therapy strategy than adjustment of the pressure level of the valve. Patients of the “secondary non-responder” group completed in only 27% all three steps of shunt management. We were able to reduce the rate of secondary deterioration from 20 to 15% with our shunt management. This leads to an improvement in the long-term outcome of iNPH patients. However, it cannot be ruled out that more consistent use of our proposed algorithm could further minimize the non-responder rate.

### Shunt management

Kahlon et al. [24] showed in the long-term follow-up study of patients with clinical symptoms of NPH, that only 20% of these patients continued clinical improvement after 5 years. A criticism of the study would be that not all patients completed the long term follow up because of a high mortality rate of 37%. Moreover, it is not mentioned whether shunt revision were performed to improve the symptoms [24].

Toma et al. [30] reviewed 64 studies with 3063 patients concerning the outcome of shunt surgery in patients with NPH. The author concluded that the benefit of VPS is long lasting, but requires frequent shunt revisions. Pujari et al. [31] revealed, that over 80% of his patients showed an improvement in the follow up examination after 7 years and 53% required multiple shunt revisions. Other studies reported a shunt failure rate up to 32% [32, 33]. However, Reddy et al. [33] noticed, that the majority of the shunt revisions occur in the first 6 months after VP-shunting. Regarding our results, a surgical shunt revision is not necessary in all cases of secondary deterioration to improve the symptoms. Adjusting the valve pressure level or shuntography may be sufficient. In cases where neither results in the desired therapeutic effect we recommend to replace the fixed ASD with an adjustable ASD (see Fig. 2). Kehler et al. [34] verified in a prospective registry study, that the implantation of an adjustable ASD (proSA) adds no further risk and results in improvement of the hydrocephalus symptoms in 55% of the patients. Furthermore, the author added, that the proSA gives the opportunity for several adjustments in order to find the appropriate setting for the patient [34]. This suggests adjustable gravitational units should be implanted at the time of the first surgery. Before the start of recruitment in the SYGRAVA [35] trial, we did not implant adjustable gravitational units in the first surgery due to missing prospective trial data. This prospective trial is being conducted on the subject of efficiency and safety of adjustable gravitational units [35].

### Risk factors

Another focus of our study was to determine risk factors for the development of “secondary non-responder”. The “secondary non-responder “group was significantly older than the “shunt insufficiency” group (68 years vs. 73 years). Kahlon et al. [24] investigated in the long-term evaluation of patients with NPH and VPS, found that 64% of the younger patients (mean 66.7 years) continued to show improvement in the walk tests, with only 11% of the older patients (mean 74.7 years) continuing to improve [24].

It can be assumed that the patient’s age increases the likelihood that further neurodegenerative diseases (Alzheimer’s disease, dementia with Lewy bodies, Parkinson disease, progressive supranuclear palsy) may occur. As in our study, neurodegenerative diseases are frequent causes of poor outcome after VP-shunting [22]. The extrapyramidal symptoms of these diseases can mimic the symptoms of patients with NPH [36]. Even these patients can improve temporarily after VP-shunting [10]. It cannot be excluded, that some of our shunted patients were in the preclinical phase of the neurodegenerative disease at the time of surgery. Thus, it is essential to rule out meticulously the disease before VPS placement. Junkkari et al. [37] highlighted in the recently published study, that an accurate identification of NPH has a huge impact on the results of our treatment. Consequently, a systemic diagnostic workup becomes indispensable [37]. Espay et al. [38] goes one step further and postulates that the NPH should be a diagnosis of exclusion that requires careful consideration of neurodegenerative disorders [38]. But he also concluded in the study, that VP-shunting may remain a reasonable option for short-term management of patients with neurodegenerative disorders [38]. Junkkari et al. [37] points out that additional tests should not delay treatment.

A further significant risk factor in our study seems to be clinical and radio-morphological signs of overdrainage. Overdrainage only occurred in the group of “secondary non-responders”. This could be explained by bias in the definition of both groups, which consequently places the classification of these patients in favor of the group with “secondary non-responder”. The therapeutic aim with overdrainage is to reduce the CSF flow rates in the VPS by adjusting the valve setting to higher pressure levels. In our department we first increase the opening pressure setting to a minimum of 100 mmH_2_O. Alternatively, we replace the fixed gravitational unit with an adjustable gravitational unit.

### Limitations

In our Department all patients with the suspected iNPH were diagnosed following a standardized diagnostic pathway including intrathecal infusion test and CSF tap test. Nevertheless, this pathway may not be able to completely exclude patients with other neurodegenerative disorders mimicking NPH symptoms. Unfortunately, new neurological diseases were screened out of the medical records available in our hospital and external medical records are usually not accessible in the case of treatment outside of our hospital. As this is a retrospective study, it cannot be ruled out that, over such a long period (1999 to 2013), clinical decisions that could influence the results have changed. Our proposed algorithm for shunt management is based on one center results without prospective randomized evaluation and was developed for standardization of therapeutic decisions in the event of secondary deterioration. Therefore, we cannot exclude that the group of “secondary non-responder” included some patients with “shunt insufficiency”. The next aim is to evaluate our algorithm in prospective manner in order to have statistical validity. The quality of evidence of the algorithm corresponds to level III. Furthermore, given the dropout rate of 15%, it cannot be excluded that these patients could no longer complete follow-up due to clinical worsening.

## Conclusion

Twenty percent of a large cohort of patients who initially benefitted from VPS for iNPH developed “secondary deterioration” 2.7 years after surgery. Due to several actions of shunt management following an algorithm, about one quarter of these patients recovered the initial benefits.

Higher age at the time of shunting, newly diagnosed neurodegenerative diseases, and overdrainage requiring adjusting the valve to higher pressure levels might be risk factors for becoming “secondary non-responder”. These findings underline the importance of long-term follow-ups and lifelong care to achieve favorable outcomes for patients with iNPH. In the case of secondary deterioration, a complex therapy management including invasive methods should be considered.

## Data Availability

The datasets used during the current study are available from the corresponding author on reasonable request.

## Abbreviations

iNPH: Idiopathic normal pressure hydrocephalus
NPH: Normal pressure hydrocephalus
sNPH: Secondary normal pressure hydrocephalus
VPS: Ventriculoperitoneal shunt
CSF: Cerebrospinal fluid
KS: Kiefer score
cCT: Cerebral computer tomography
ASD: Anti-siphon device
F/U: Follow-up
CMI: Comorbidity index
